# An Efficient Method for the Euthanasia of Cane Toads (*Rhinella marina*) under Northern Australian Field Conditions

**DOI:** 10.3390/ani11082239

**Published:** 2021-07-29

**Authors:** Winston R. Kay, Peter R. Mawson

**Affiliations:** 1 Species and Communities Branch, Department of Biodiversity, Conservation and Attractions, South Perth, WA 6151, Australia; 2 Perth Zoo Science, Department of Biodiversity, Conservation and Attractions, South Perth, WA 6151, Australia

**Keywords:** euthanasia, cane toad, *Rhinella marina*

## Abstract

**Simple Summary:**

Cane toads are a highly invasive species that present a serious threat to most vertebrates that eat them. In Australia, their spread from the point of introduction in 1935 has seen them occupy any suitable habitat across the north of the country, with the potential to be transported to locations in southern Australia. Control and safe removal of unwanted toads presents ethical and occupational health risks to those engaged in such activities. This study describes an efficient method of control suitable for application under field conditions anywhere in Australia.

**Abstract:**

The euthanasia of cane toads under field conditions presents a number of logistical and animal welfare challenges. One recommended method of control involves the use of carbon dioxide in plastic bags. This paper describes the minimum amount of time (4 h) required to efficiently euthanase toads with a carbon dioxide concentration of 4.96% under field conditions experienced in northern Australia. Discussion is also provided on the issues of safe disposal of biological and plastic waste associated with the application of this method.

## 1. Introduction

The cane toad (*Rhinella marina*) is amongst the 100 worst invasive alien species [1]. The species was introduced into Australia in 1935 to help control two species of cane beetles affecting Queensland cane crops. From the initial point of release at Gordonvale, Queensland, the species spread across much of Queensland, parts of northern New South Wales, northern parts of the Northern Territory and in 2009 entered north-eastern Western Australia. Predictive modelling based on climate matching suggests that the cane toad could occupy most of the coastal parts of Western Australia as far south as the south-west capes region [2,3].

In the 20 years to 2011, the State, Territory and Australian governments have invested more than AUD 17 million in researching ways of mitigating the impact that cane toads have on the natural biodiversity of Australia [4]. To date, no effective means of combating the spread or impact of cane toads have been identified. At a local level, community-based groups have been organized to physically remove cane toads from selected areas and to then euthanase them. This action has received considerable publicity as well as public funding [5].

These well-organized community activities received media coverage and support from a broad section of the community, including individual State, Territory and Federal politicians. However, a disturbing aspect of some of those media articles has been the methods that were advocated for killing cane toads. These ranged from the use of sporting equipment to standing on toads or applying a range of chemicals that can be regarded as caustic, acidic or toxic. Household disinfectant products have also been championed as being suitable for killing cane toads [5].

The scientific and animal welfare literature offers further choices, including chemicals such as MS222 (tricaine methane sulphonate), potassium chloride, chloral hydrate, pentobarbitone (Nembutal^®^ or Lethabarb^®^), benzocaine and various alcohol-based products [6,7]. Shine et al. [8] provided empirical evidence in a study of cane toads that cooling followed by freezing in amphibians may be humane, although most euthanasia guidelines recommend against this technique [6,9].

More recently, ANZCCART [10] published guidelines for the humane killing of cane toads, which included a number of methods such as commercial topical sprays, refrigeration followed by freezing, use of clove oil then freezing, stunning and decapitation and gassing with CO_2_ followed by deep burial. All of these methods were considered ‘acceptable with reservations’, but no information was provided as to the nature or extent of those reservations or the process by which those recommendations were reached. This ‘eminence-based’ approach [11] is antiquated and contributes little to improving animal welfare outcomes [12,13]. In the discussion section of the ANZCCART [10] paper, it is acknowledged that a major problem when evaluating various methods of killing cane toads is the lack of empirical evidence. The author(s) of the ANZCCART guidelines [10] goes on to suggest that any future testing of agents needs to be undertaken as a matter of priority and include properly constructed dose–response trials. The AVMA [14] provides advice on acceptable and unacceptable methods for euthanasing amphibians.

One of the more recent and extensively used methods employed by community groups in the Northern Territory, and which was advocated for use in Western Australia (WA), was the inhalation of carbon dioxide gas (CO_2_). This paper provides the empirical evidence that informed the decision by the WA government to authorize the use of inhalation of CO_2_ followed by deep burial for the euthanasia and bulk disposal of cane toads under northern Western Australian field conditions.

## 2. Materials and Methods

### 2.1. Pilot Studies

In preparation for the inevitable arrival of cane toads in WA, the Department of Environment and Conservation (DEC; now the Department of Biodiversity, Conservation and Attractions (DBCA)) developed a Cane Toad Management Strategy [15] and examined options for the humane killing of cane toads. Preliminary research undertaken by the DEC in 2005 confirmed that a range of chemicals (potassium chloride solutions 5% *w*/*v* and 10% *w*/*v* injected intra-thoracic; topical application of ethanol, lignocaine, benzocaine and a proprietary brand disinfectant) were either ineffective in killing cane toads or took more than 10 min to kill them. Gas inhalation (14 L/min of CO_2_ applied for 20, 40 or 80 min, after which the toads were removed from the CO_2_ and placed in ambient air and monitored for signs of recovery) was also ineffective. Physical methods based on blunt trauma to the brain, achieved by pithing, double pithing and destruction of the brain with nail guns modified so that the striking pins delivered the lethal trauma rather than nails, successfully and humanely killed cane toads under laboratory conditions. However, the DBCA considered none of the methods trialled particularly useful under northern Australian field conditions where the number of toads that would need to be euthanased could number in the hundreds or thousands in any one 24-h period. The reasons for these methods being deemed unsuitable included the occupational, health, safety and welfare of the operator under field conditions; regulatory requirements (for the use of scheduled poisons); logistic difficulty in delivering the method in the field; and cost (or combinations of two or more factors). One of the methods that was investigated in the DEC’s 2005 trials (CO_2_ inhalation) was subsequently applied in field control operations conducted by non-government organizations operating in the Northern Territory.

Amphibians, and cane toads in particular, have well-developed physiological capacities to survive in low oxygen or anoxic environments [16]. In addition, they have physiological capabilities to deal with increasing levels of CO_2_ in the blood stream and can shunt CO_2_ into other tissues for short-term storage or excrete it via their skin [17]. Coelho and Smatresk [17] showed that not only do cane toads have these physiological capacities, but they also use simple behavioural responses, such as breath holding (for up to 8 h), to minimize their exposure to CO_2_. When these factors were considered along with the results from the DEC’s earlier research, the DEC had concerns about the suitability of CO_2_ inhalation to deliver a humane and completely reliable means of euthanasing cane toads under field conditions in Western Australia.

On this basis, it was considered necessary to determine whether the field methods used by community groups in other jurisdictions during the period 2007–2010, and which were proposed for use in Western Australia, would result in (1) the humane death of toads subjected to inhalation of CO_2_, and to define how long it would take for 100% of the subjected toads to die (Experiment 1); and (2) determining whether confining groups of toads of varying size in plastic bags influenced the capacity of CO_2_ inhalation to either kill toads outright or to alter the time to death (Experiment 2).

### 2.2. Experimental Regime

Two experiments were conducted during the daytime under controlled conditions at ambient temperatures that simulated the night-time temperature range (25–30 °C) experienced in the field in the Kimberley region of Western Australia. Rooms with air conditioning were available, if necessary, to ensure that ambient temperatures during the experiments did not fluctuate outside the desired range. All cane toads used in this study were kept in full and permanent shade during captivity and the course of the experiments. The cane toads were captured during a single night and transported in locked boxes by road in air-conditioned vehicles to WA early the following day.

Experiment 1: Juvenile and adult toads were placed individually in sealed plastic bags (27 L, 270 mm × 510 mm commercially available brand of clear plastic kitchen tidy bags of the type normally used by the local community groups undertaking cane toad reduction operations) filled with either CO_2_ (treatment) or ambient air (control). All toads were inspected before being placed into the bags to ensure that they had not sustained any injuries during capture and transported to Kununurra (−15.7736° S, 128.7386° E) from the Northern Territory (under WA Department of Primary Industries and Regional Development permit #496) by road in an air-conditioned vehicle, and that they were alert, mobile and therefore suitable to participate in the experiments.

Treatment 1: Three adult toads (>90 mm snout-vent length (SVL)) were placed in each of 19 bags filled with CO_2_ (*n* = 57 toads) from a compressed CO_2_ cylinder until a CO_2_ concentration of 4.96% was achieved (CO_2_ concentration in ambient air is 0.04%). The necks of the bags were twisted closed, folded over at the top and secured with a cable tie. A matching series of controls were placed in bags filled with normal air (*n* = 57 toads) and sealed in the same manner. Three toads were placed in each bag to give some measure of individual variation in response to exposure to CO_2_, and to being confined in a bag with normal air. The clear plastic bags allowed direct observation of the toads while they were confined.

At intervals of 0.5 h, and then at hourly intervals from 1 h to 18 h, all three toads were removed from one bag each from the treatment and control groups and placed on their backs in separate, labelled containers (175 × 115 × 60 mm) with holes drilled in the lids and lined with moist paper towel on the bottom and with free access to ambient air. Any attempt the toads made to assume the normal sitting position provided clear evidence of the recovery. Each toad was monitored for up to 6 h after removal from the bag containing CO_2_ to determine whether it had died or recovered, or to determine whether confinement in a plastic bag containing air adversely affected the toads (Control group). Observations were made on the behaviour of each toad (active or inactive), its position (sitting in normal position, or lying on its back), colour of the belly skin (pale white/grey or pink) and any indication of breathing (buccal or rib movement) were noted on a data sheet maintained for each toad.

This experimental regime was used to determine whether death by hypoxia or some other factor related to placing toads in sealed plastic bags occurred before death by CO_2_ exposure, or the reverse. If CO_2_ was acting as an anaesthetic agent [14], it may facilitate a more rapid death than one where toads were simply deprived of oxygen.

If all the toads that were exposed to the CO_2_ treatment regime recovered, then there was no merit in any further testing. If, however, toads died from CO_2_ exposure after a period of exposure, then a second experiment was required to confirm whether toads would be killed under conditions replicating those employed in the field, whereby toads are placed in bags in large groups.

Experiment 2: Adult toads were placed in groups of varying size (as described below) in sealed plastic bags (27 L, 270 mm × 510 mm, clear plastic kitchen tidy bags), which were then filled with CO_2_ as described above. All toads were examined to ensure no prior injuries, as described above.

Treatment 1: 5 adult toads in each of 10 bags filled with CO_2_ (*n* = 50 toads);
Treatment 2: 10 adult toads in each of 10 bags filled with CO_2_ (*n* = 100 toads);
Treatment 3: 20 adult toads in each of 10 bags filled with CO_2_ (*n* = 200 toads).

Once the toads had been sealed in the bags containing CO_2_, they were monitored hourly until the expiry of the time to death determined in Experiment 1. Toads were then removed, and each toad placed in a separate, labelled container with moist paper towel on the bottom and free access to normal air. Each toad was monitored for up to six hours after removal from the bag containing CO_2_ to determine whether it was dead or had recovered. The proportion of toads that died was calculated at the end of the six-hour recovery period.

Any toads that remained alive at the end of the experiment were euthanased using blunt trauma to the brain, as described above. Euthanasia of any toads that remained alive at the end of the experiments was a permit condition set by the regulating authority for the keeping of cane toads in Western Australia.

### 2.3. Ethical and Import Approvals

The experiments were conducted at the Department of Environment and Conservation offices in Kununurra and were approved by the DEC Animal Ethics Committee (CALM AEC 15/2005 and DEC AEC 12/2010). The experiments were conducted on 29–30 June 2010. Importation of live toads from the Northern Territory was approved under the Western Australian Department of Agriculture and Food WA import permit No. 496.

## 3. Results

Observations of the toads through the plastic bags indicated that all animals settled quickly once the experiments began. The toads in the bags filled with air moved sparingly, while those in the bags filled with CO_2_ all drew their limbs in close to their bodies and remained still until they were removed from the bags at the pre-determined times.

Cane toads exposed to CO_2_ for 0.5 h were rendered unconscious, did not respond to any tactile stimulus applied to their toes, showed no signs of breathing (buccal or rib movement) but had observable heartbeats. When left to recover, they were all able to right themselves when removed from the bags. One toad was observed to resume a sitting position after only 18 min and the other two toads did so after 34 min (Table 1). At the time they were removed from the CO_2_, two of the toads had pale grey-coloured bellies and the third showed evidence of some red colouration on the belly skin. All three toads made full recoveries, and 6 h after removal from the CO_2_ were active and alert. All three toads in the control group confined for 0.5 h to the bag filled with air were active and alert from the time they were removed from the bag and remained so later.

Similar results were observed in the toads exposed to CO_2_ for 1 h, with all toads being unconscious when first removed from the bag, not responding to tactile stimulus and one toad having pink belly skin. Heartbeats were evident and the toads were observed to resume a sitting position 31–45 min after removal from the CO_2_. All three toads recovered and were mobile and alert 6 h after removal from the CO_2_. None of the toads in the control group confined to the bag for 1 h showed any adverse effects from the confinement.

After 2 h exposure to CO_2_, two of the three toads were dead and rigor mortis had set in. The third toad was unconscious, non-responsive to tactile stimulus, had pale coloured belly skin but an observable heartbeat. This animal remained in this state for the next six hours and never recovered and was subsequently euthanased. None of the toads in the control group confined in the air-filled bag for 2 h showed any adverse effects and were mobile and alert from the time they were removed from the bag and remained so 6 h later.

After 3 h exposure to CO_2_ all three toads were dead, with two toads showing signs of rigor mortis. Two toads also had some pink colouring to the belly skin. None of the toads confined to the air-filled bag for 3 h showed any adverse effects from their confinement and were mobile and alert on release from the bag and remained so 6 h later.

Similar results were observed of the toads exposed to CO_2_ and air for longer periods of time ranging from 4–10 h, with the exception that the toads that had died began to bloat and show early signs of decomposition. All toads in the control groups remained mobile and alert. These results indicate that a minimum exposure time of four hours to CO_2_ is required to ensure 100% mortality of cane toads. Based on these results, the second experiment described in the Methods was conducted, with the toads subjected to 4 h exposure to CO_2_. All toads in the groups of 5, 10 and 20 exposed to the CO_2_ were dead when removed from the CO_2_ filled bags after 4 h exposure.

## 4. Discussion

Our study showed conclusively that inhalation of CO_2_ provided at a concentration of 4.96% can kill cane toads exposed for 4 h. To ensure that 100% of the treated toads are killed, the exposure times should be extended to 4 h. Within the range of this study (5–20 individuals), the number of toads confined to the standard 27 L bags does not influence the mortality rate, provided that the exposure time is at least 4 h. Based on these results, the DEC approved the use of CO_2_ inhalation for the euthanasia of cane toads under field conditions in Western Australia in August 2010.

The clear plastic bags allowed direct observation of toads that were exposed to CO_2_ or air, and while none of the toads exposed to CO_2_ showed any obvious behaviour that would indicate distress, it cannot be assumed that none was caused.

The appeal of the utility of this method is obvious, in that it provides a simple means of euthanasing large numbers of toads in the field. However, care would need to be taken to ensure that environmental conditions, such as high ambient daytime temperatures, did not lead to distress of toads while CO_2_ inhalation was taking effect. This may not be a major problem as most community operated toad collection programs in Australia occur at night. Given that most euthanasia guidelines that advocate the use of CO_2_ for amphibians refer to its application to individual animals rather than its use for batches of animals, and in the absence of further field trials, the number of toads placed in each bag should be limited to 20 to ensure that the concentration of CO_2_ remains high enough to guarantee a humane death and that the mass of toads in the bags does not in itself became a factor influencing animal welfare before death by inhalation of CO_2_. Further testing will be required before the limit on the number of toads that are placed in each bag is increased.

The only potential problem associated with this method is what to do with the plastic bags after the dead toads have been removed and buried. It is possible that during the early stages of confinement that the toads may have released quantities of bufotoxin, which may present an occupational health risk to the field staff engaged in the toad control operations. Safe disposal or re-use of intact bags will help reduce the potential for non-target fauna coming in contact with contaminated bags through scavenging. From an occupational safety perspective, the neurotoxic and cardiotoxic effects attributed to bufotoxin envenomation in humans arise following ingestion of the poison [18,19], rather than contact with the skin. However, given the remote locations where cane toad control activities often occur, and that access to medical treatment may not be possible in a timely manner, a cautious approach would seem warranted.

Based on the results from this study there is now clear evidence to support the use of cooling followed by freezing [20,21] and exposure to CO_2_ gas for periods longer than 4 h (this study). The CO_2_ method is likely to be preferred for use in northern Australian field conditions, where freezers may not be available. For locations where freezers are available, the cooling-then-freezing method may be preferred as it would not require access to a commercial supply of CO_2_.

## Figures and Tables

**Table 1:** Effect of the increasing duration of exposure to CO_2_ on the state of consciousness and capacity to fully recover when returned to ambient air for six hours. Sample size for each exposure time: *n* = 3 adult male cane toads.

Response	Exposure Time (h) to CO_2_				
	0.5	1	2	3	4
No. unconscious *	3(3)	3(3)	1(0)	0	0
No. dead	0	0	2	3	3
No. moribund	0	0	1	0	0

* Animals were defined as unconscious if on removal from the CO_2_ environment they showed no spontaneous movement or righting response and were not breathing. Numbers in parentheses indicate the number of unconscious animals that recovered alertness and mobility 6 h post removal from CO_2_.

## Data Availability

Data is contained within the article.
